# Goodyear Dental Vulcanite Co.

**Published:** 1881-03

**Authors:** 


"THE GOODYEAR DENTAL VULCANITE CO."—We have a number of letters from dentists throughout the country in reference to this company and what will come of the Cummings Patent. We would gladly publish all of them but space will not permit. We will state that we haven't the remotest idea that this company will attempt to have said patent extended by Congress, as there is not a shadow of chance for success. The company has reaped a *large* annuity from the dental profession in the United States, and we think, are willing to retire from the contest after June 7th, 1881, and let us use rubber *ad libitum*. The courts give us the right to use celluloid.

